# Recent advances in the mechanisms of PD-L1 expression in gastric cancer: a review

**DOI:** 10.1186/s40659-025-00597-3

**Published:** 2025-03-17

**Authors:** Peifeng Chen, Zhangming Chen, Wannian Sui, Wenxiu Han

**Affiliations:** Department of General Surgery, First Affiliated Hospital of Anhui Medical University, No. 218 Jixi Avenue, Shushan District, Hefei, Anhui Province 230022 China

**Keywords:** Gastric cancer, Programmed death ligand 1, Tumor immunity, Tumor microenvironment

## Abstract

In the progression of gastric cancer (GC), various cell types in the tumor microenvironment (TME) exhibit upregulated expression of programmed death ligand 1 (PD-L1), leading to impaired T-cell function and evasion of immune surveillance. Infection with *H. pylori* and EBV leads to increased PD-L1 expression in various cell types within TME, resulting in immune suppression and facilitating immune escape of GC cells. In the TME, mesenchymal stem cells (MSCs), M1-like tumor-associated macrophages (MI-like TAM), and myeloid-derived suppressor cells (MDSCs) contribute to the upregulation of PD-L1 expression in GC cells. Conversely, mast cells, M2-like tumor-associated macrophages (M2-like TAM), and tumor-associated neutrophils (TANs) exhibit elevated levels of PD-L1 expression in response to the influence of GC cells. Together, these factors collectively contribute to the upregulation of PD-L1 expression in GC. This review aims to provide a comprehensive summary of the cellular expression patterns of PD-L1 in GC and the underlying molecular mechanisms. Understanding the complex regulatory pathways governing PD-L1 expression may offer novel insights for the development of effective immunotherapeutic interventions.

## Introduction

Gastric cancer (GC) is the fifth most common cancer and the third most common cause of cancer death globally [1]. The incidence and mortality rates of GC are significantly higher in certain regions, particularly East Asia, Eastern Europe, and South America. While developed nations have experienced a decline in GC incidence rates, the demographic aging of populations may potentially lead to future escalations in the prevalence of this disease [2]. Infection with *Helicobacter pylori* (*H. pylori*) is a significant risk factor for non-cardia GC. Eradication therapy for *H. pylori* has been shown to reduce the risk of GC [3]. In addition to *H. pylori* infection, various factors contribute to the risk of GC, including age, socioeconomic status, smoking, alcohol consumption, family susceptibility, history of gastric surgery, and malignancy-associated anemia. Adopting preventive measures such as reducing salt and salty food intake, along with increasing the consumption of fruits and vegetables, can play a significant role in lowering the risk of GC [4].

Surgery remains the cornerstone of therapeutic interventions for GC, providing the most effective means of tumor therapy [5]. However, for patients who are deemed ineligible for surgical intervention due to advanced disease stage, comorbidities, or other factors, tumor immunotherapy have emerged as promising avenues [6]. Among these modalities, PD-L1 (programmed cell death ligand 1) and PD-1 (programmed death 1) inhibitors have demonstrated promising anti-tumor immune effects in immunotherapy [7]. The PD-1 gene, also known as cluster of differentiation 279 (CD279), can be found on chromosome 2q37 [8]. The expression of PD-1 suppresses various immune cell subsets in the TME, including T cells, B cells, natural killer cells, macrophages, and dendritic cells [9–12]. When PD-1 binds to its ligand PD-L1, it inhibits the activity of T cells, allowing tumor cells to escape immune surveillance [13].

A significant body of clinical research has confirmed the benefits of combined PD-1 immune checkpoint inhibitors and chemotherapy in the treatment of GC. For instance, the CheckMate 649 trial compared nivolumab combined with chemotherapy to chemotherapy alone. The results demonstrated a significant improvement in overall survival with nivolumab plus chemotherapy. And nivolumab plus chemotherapy has become the new standard first-line treatment for previously untreated patients with advanced gastric, gastro-esophageal junction, or esophageal adenocarcinoma [14]. Furthermore, the ORIENT-16 trial compared the overall survival of patients receiving sintilimab plus chemotherapy to those receiving placebo plus chemotherapy. In patients with unresectable locally advanced or metastatic gastric and gastro-esophageal junction adenocarcinoma receiving first-line chemotherapy, sintilimab significantly improved the overall survival for all patients as well as those with a PD-L1 combined positive score (CPS) of 5 or more when compared to placebo [15]. These findings collectively demonstrate the substantial clinical efficacy of PD-1 immune therapy in combination with chemotherapy for GC, offering promising prospects for the treatment of this malignancy.

This article aims to provide a comprehensive review of various factors and related mechanisms that contribute to the upregulation of PD-L1 expression in GC. By understanding these factors and mechanisms, we can gain insights into potential therapeutic targets that help overcome immune escape in GC and improve treatment outcomes.

## Pathogen

### Epstein-Barr virus (EBV)

EBV is a member of the herpes virus family, which is associated with the development of GC. The Cancer Genome Atlas (TCGA) proposes the four molecular classifications of GC: EBV-positive, microsatellite instability (MSI), genomically stable and chromosomal instability [16], and the EBV-associated GC (EBVaGC) accounts for about 10% [16]. In a prospective phase 2 clinical trial, the overall response rate of pembrolizumab is observed as 100% in all EBV-positive metastatic GC [17]. Moreover, EBVaGC displayed the higher expression of PD-L1/L2 [18–20], extensive MSI and aberrant CpG hypermethylation [21, 22], and rare TP53 mutations [23].

**Fig. 1:**
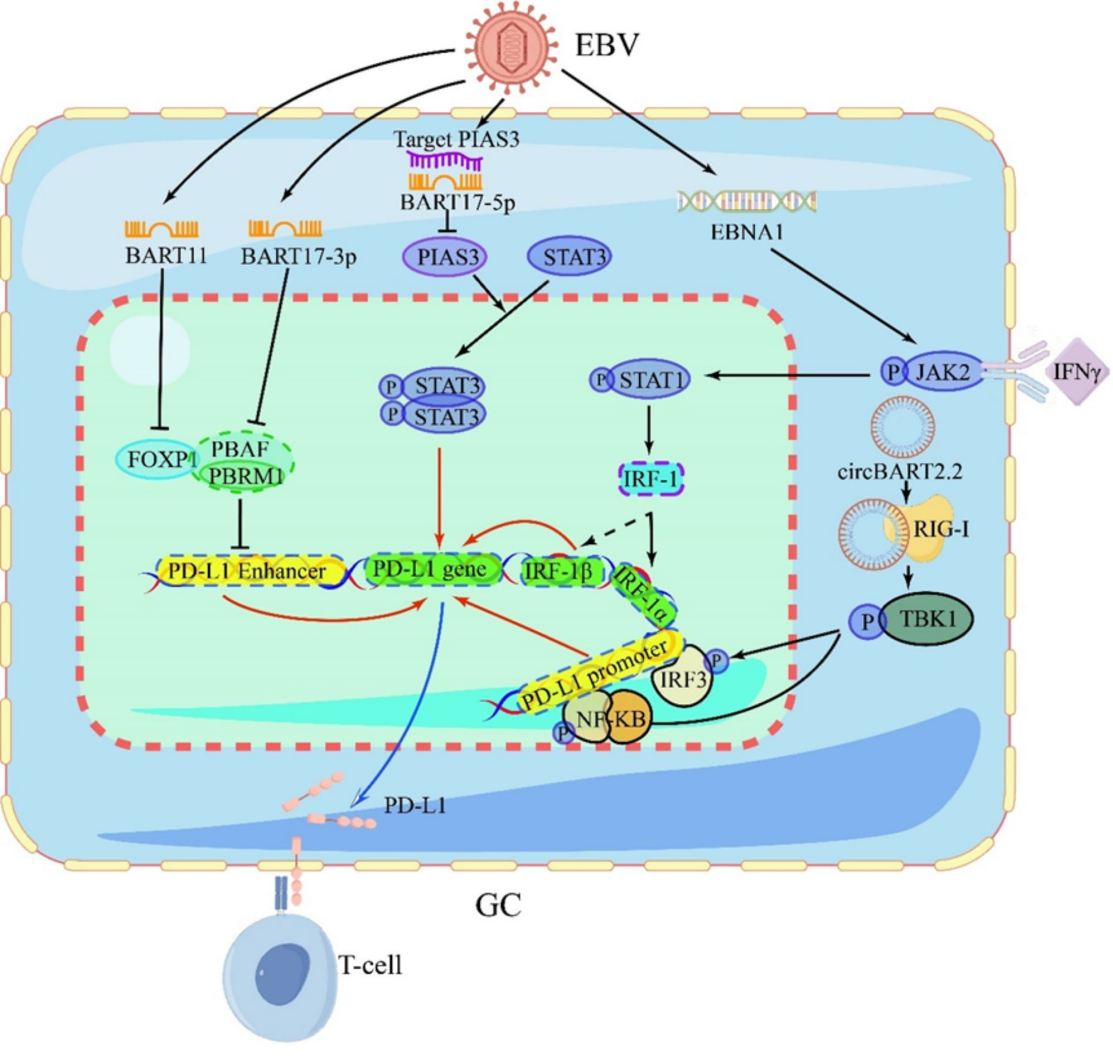
EBV involves in regulating the PD-L1 expression and immune escape

Recently, researchers pay more attention to the EBV mediated immune escape in GC. Zeng and colleagues found that 2 of 44 EBV-coding-miRNAs upregulates the expression of PD-L1, leading to the T cell apoptosis and tumor immune escape [24]. EBV-miR-BART11 decreases the expression of FOXP1 by binding directly to the 3′-UTR of FOXP1 mRNA, which removes FOXP1 mediated inhibition of PD-L1 expression, eventually leading to upregulation of PD-L1 [24]. Meanwhile, EBV-miR-BART17-3p also promoted PD-L1 transcription by targeting PBRM1 [24]. Besides, EBV-miR-BART5-5p decreases the expression of PIAS3, which abolishes the PIAS3 mediated inhibition of STAT3 phosphorylation, resulting in the upregulation of PD-L1 finally [25]. EBV nuclear antigen 1 (EBNA1), expresses in all EBVaGC cells and functions as a transcriptional factor, regulates expression of EBV genes and host cellular genes [26, 27]. In three EBV (+) GC cell lines (SNU-719, YCCEL1 and NCC-24), both proteins and mRNAs of PD-L1 were positively correlated with EBNA1 levels. In vitro, EBNA1 cooperated with IFN-γ activates the JAK2 / STAT1 / IRF-1 signaling pathway, which promotes IRF-1 mediated transcription of PD-L1 by binding to its promoter (-171 ~ -159) [28]. Meanwhile, infection of EBV also increases the IFN-γ production by activating IRF3 [29]. EBV-encoded small RNAs (EBERs) are recognized by Retinoic acid-inducible gene I (RIG-I), which initiates IRF3 and NF-κB signaling pathways, leading to induction of IFNs [30]. Zeng et al. found that EBV-encoded circBART2.2 upregulates PD-L1 expression by promoting IRF3 and NF-κB to bind with PD-L1 promoter (Fig. 1) [31]. Hence, it is still extremely challenging in elucidating mechanism about regulating PD-L1 expression, mediating immune escape and reacting to immune checkpoint inhibitors in EBVaGC.

EBV-encoded BART11 or BART17-3p target the transcription of FOXP1 or PBRM1, resulting in transcriptional repression and downregulation of their expression. PBRM1 forms the PBAF complex and interacts with FOXP1 to inhibit the PD-L1 enhancer, leading to the downregulation of PD-L1 expression and promoting immune escape in tumors; The EBV-encoded miR-BART5-5p directly targets PIAS3, leading to the downregulation of PIAS3 protein levels and subsequent activation of STAT3. Phosphorylated STAT3 translocates into the cell nucleus and promotes the transcription of the PD-L1 gene, resulting in the upregulation of PD-L1 levels; Upon IFNγ stimulation, EBNA1 upregulates JAK2 expression and activates the JAK2/STAT1/IRF-1 signaling pathway, enhancing PD-L1 expression; EBV-encoded circBART2.2 upregulates PD-L1 expression by facilitating the binding of IRF3 and NF-κB to the PD-L1 promoter.

### Helicobacter pylori (H. pylori)

*H. pylori* is a gram-negative, microaerophilic, and flagellated bacteria, and is also major cause of GC, especially for 90% non-cardia GC [32, 33]. Recently, Paul et al. found that *H. pylori* seropositivity decreases the effectiveness of anti-CTLA4/PD-L1 combination therapy in clinical cohorts of colon cancer and non-small cell lung cancer [34].

**Fig. 2:**
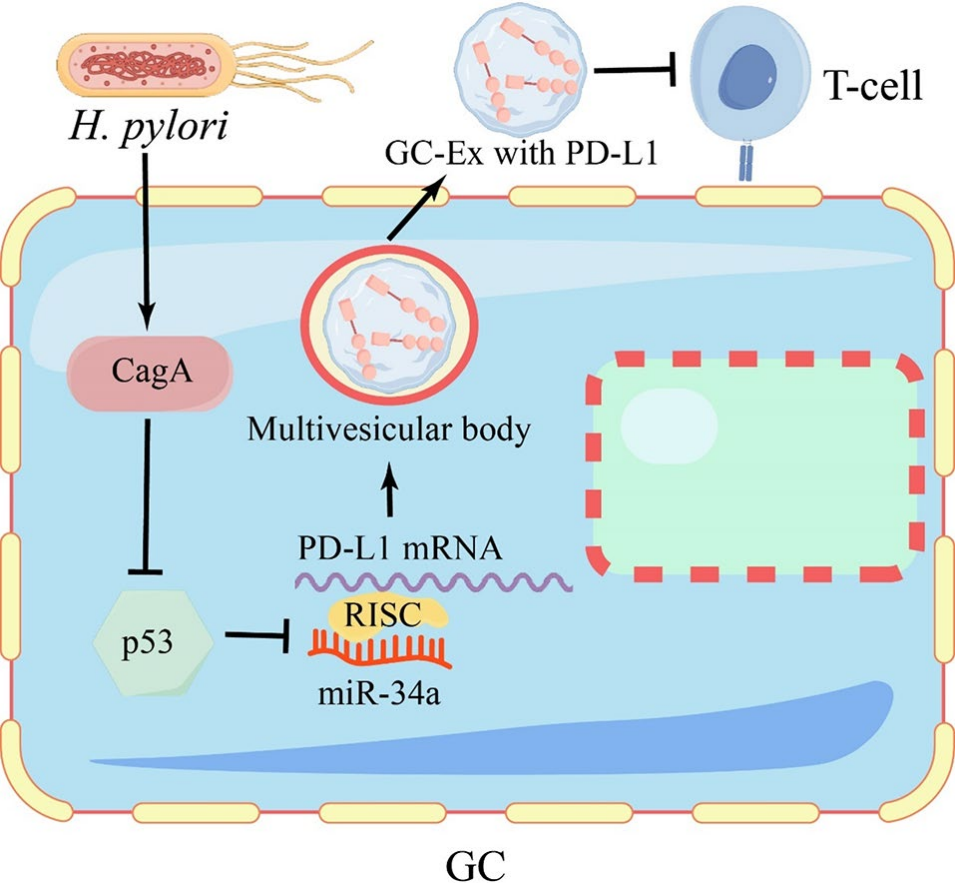
*H. pylori* infection increases PD-L1 expression in GC cell

In *H. pylori* -infected gastric tissue, high levels of PD-L1 expression are positively correlated with the progression of pre-cancerous gastric lesions and GC [35–37]. Cytotoxin-associated gene A (CagA), a virulence factor of Helicobacter pylori, plays a pivotal role in the carcinogenic process of GC by activating tumor signaling pathways [38]. CagA can activate AKT and ERK to phosphorylate human double minute-2 (HDM2), leading to the dissociation of the HDM2-p53 protein complex. This activation also triggers HDM2 and ARF-BP1 E3 protein ligase, thereby promoting the rapid degradation of the p53 protein [39]. Meanwhile, p53 can promote the expression of miR-34a, while simultaneously suppressing the expression of GC cell-derived exosomes (GC-Ex) with PD-L1. Thus CagA promotes immune evasion in GC via the p53-miR-34a-PD-L1 signaling axis, thereby inhibiting the proliferation of CD8 + T cells and cytokine secretion (Fig. 2) [40].

*H. pylori*-generated CagA enters GC cells, downregulates p53 levels to reduce miR-34a levels, promotes the secretion of GX-EX carrying PD-L1 into the extracellular milieu, facilitating immune evasion in GC, inhibiting the proliferation of CD8 + T cells and cytokine secretion.

However, there has been limited research investigating the role of *H. pylori* infection in carcinogenesis via regulating PD-L1. Previous investigations have largely focused on how *H. pylori*-induced gastritis and pre-cancerous lesions promote PD-L1 expression. Thus, we will review some of the key findings from the studies to propose the potential mechanistic role of this pathway in the development of GC.

**Fig. 3:**
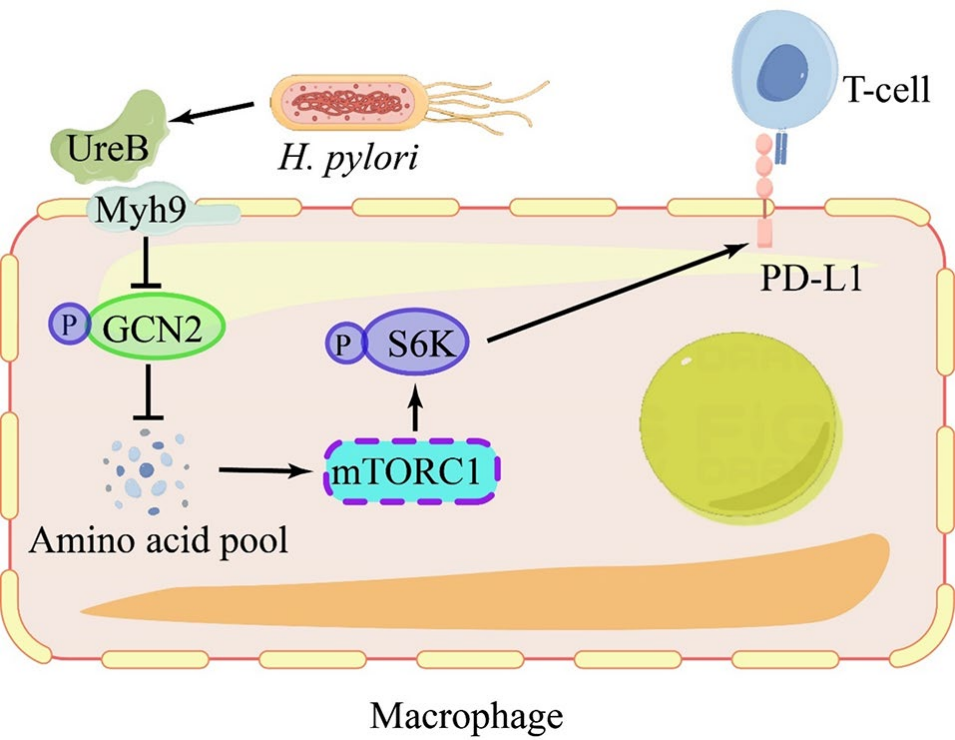
*H. pylori* infection increases PD-L1 expression in macrophage

*H. pylori* infection-induced inflammation elicits a PD-L1 response, distinct from non-infectious chronic inflammation, which inhibits the activity of recruited T cells, thereby promoting immune evasion and exacerbating the infection [41]. The establishment and colonization of *H. pylori* infection rely on a crucial virulence factor known as urease. This multimeric enzyme is composed of 12 urea molecules and UreB heterodimers, which plays a vital role not only in the initial establishment of infection but also in the maintenance of chronic infection [42]. The interaction between UreB and Myh9 on the macrophage membrane reduces autophosphorylation of GCN2, enhancing the intracellular amino acid pool and activating downstream mTORC1, leading to upregulation of S6K phosphorylation. Ultimately, this results in the expression of PD-L1 on macrophages, which serves to inhibit the activation of CD8 + T cells [43]. *H. pylori-*infection also leads to the activation and aggregation of eosinophils in the microenvironment. However, eosinophils show high levels of PD-L1 expression in this bacterial infection, which to some extent counteracts the eosinophil-driven T cell suppression (Fig. 3) [44].

Research using mouse models has revealed that *H. pylori* induces the activation of STAT1 and the expression of PD-L1 within gastric epithelial cells through an immune cell-dependent mechanism. This activation can impede immune surveillance of the gastric mucosa, potentially contributing to the progression of precancerous lesions to GC [45]. Meanwhile, *H. pylori* infection activates STAT3 in dendritic cells (DCs), impairing their maturation and suppressing the secretion of the pro-inflammatory mediator IL-1β [46]. Additionally, *H. pylori* orchestrates the activity-induced tolerogenic programming of DCs, enabling them to promote the peripheral conversion of regulatory T cells (Tregs) [47]. These effects ultimately facilitate *H. pylori* colonization and the establishment of an immunosuppressive microenvironment. And early evidence suggests that DCs exhibit elevated PD-L1 expression after *H. pylori* infection in vitro [48]. Recent studies have further demonstrated that *H. pylori* infection leads to the accumulation of PD-L1-expressing DCs in the gastric mucosa and submucosa of mice models [49]. During the initial phase of *H. pylori* infection, *H. pylori* triggers the upregulation of SOCS3 expression in DCs via the T4SS/TNFα/p38 signaling pathway, resulting in a reduction of PD-L1 expression. However, this effect is transient and is observed only during the early stages of infection. As the infection progresses, PD-L1 expression in DCs gradually increases [50]. In summary, multiple cell types exhibit heightened PD-L1 expression during *H. pylori* infection, enabling the bacterium’s prolonged colonization and facilitating the transition from gastritis to cancer. Eradicating *H. pylori* can suppress PD-L1 expression, protect the gastric mucosa, and impede the progression of GC.

The UreB protein produced by *H. pylori* interacts with the macrophage membrane receptor Myh9. This interaction reduces GCN2 phosphorylation and increases the amino acid pool, which activates the downstream mTORC1 signaling pathway, resulting in upregulation of PD-L1 expression.

## Tumor microenvironment (TME)

TME is a complex and dynamic ecosystem consisting of immune cells, fibroblasts, adipocytes, blood vessels, neurons, extracellular matrix, and soluble products such as chemokines, cytokines, growth factors, and extracellular vesicles. This intricate network plays a crucial role in tumor growth and progression [51]. During the initial stages of tumor development, a complex and bidirectional interaction between cancer cells and various components of the TME is established to promote cancer survival, local invasion, and distant metastasis [52]. In detail, TME can be classified into three profile: the immune-inflamed phenotype, the immune-excluded phenotype, the immune-desert phenotype [53]. The immune-inflamed phenotype is characterized by the presence of CD4^+^ and CD8^+^ T cells in the tumor parenchyma, accompanied by myeloid and monocytic cells. The immune-excluded phenotype is marked by abundant immune cells that are retained in the tumor stroma rather than penetrating the tumor parenchyma, while the immune-desert phenotype is characterized by a scarcity of T cells in both the parenchyma and tumor stroma. Clinical responses to anti-PD-L1/PD-1 therapy typically occur in patients exhibiting the immune-inflamed phenotype, characterized by a preexisting CD8 + T cell response to the tumor. In this phenotype, the regulation of anticancer immunity is often upheld through intratumoral PD-L1 expression [54]. Tumor cells expressing high levels of PD-L1 in the TME enhance immunosuppressive activity by attenuating the cytotoxicity of T cells, monocytes, natural killer cells, and macrophages [55]. Additionally, other cell types within TME such as macrophages, dendritic cells (DCs), activated T cells, and cancer-associated fibroblasts, also express PD-L1. These components collaborate to orchestrate an immunosuppressive microenvironment, aiding the tumor in evading immune detection and clearance [56].

### Mesenchymal stem cells (MSCs)

Mesenchymal stem cells (MSCs) are a cohort of nonhematopoietic cells that possess immune-modulating properties, self-renewal capacity, and multidifferential potential [57]. They have the ability to reprogram GC cells, leading to enhanced proliferation, migration, invasion, and chemoresistance [58, 59]. Numakura et al. explored the correlation between the expression of specific MSC markers (CD73, CD90, and CD105) and the clinical pathological features of GC. They identified a significant association between CD105-positive cells and poor prognosis in GC, indicating that MSCs infiltration predicts unfavorable outcomes [60].

Li Sun’s team conducted a series of sequential studies to investigate the molecular mechanisms by which MSCs regulate tumor immune function. They initially discovered that IL-8 derived from GC-derived mesenchymal stem cells (GCMSCs) could induce PD-L1 expression in GC cells. Recent studies have also identified that circ_0073453 derived from GCMSCs can regulate IL-8 expression by acting as a sponge for miR-146a-5p [61]. IL-8 derived from GCMSCs binds to CXCR1/2 on the surface of GC cells, activating the STAT3 and mTOR signaling pathways and upregulating c-Myc levels [62, 63]. The c-Myc is a transcription factor that regulates the expression of multiple gene involved in cell proliferation, growth, differentiation, and apoptosis [64]. It can transcriptionally upregulate hexokinase 2 (HK2), phosphorylating HK2 and translocating it to the nucleus. HIF-1α is a transcription factor that plays a key role in regulating gene expression in response to hypoxia. It binds to specific sequences in the DNA called hypoxia-response elements (HREs) located in the promoters of target genes. When HIF-1α binds to these HREs, it recruits other coactivators and transcriptional machinery to initiate gene transcription [65]. The nuclear translocation of HK2 forms transcriptional complexes with HIF-1α. This enhances the occupancy frequency of HIF-1α on the promoters of PD-L1 that contain the established HIF-1α-binding motif, resulting in an upregulated expression of PD-L1 [66]. MSCs can also secrete Hepatocyte Growth Factor (HGF) which is taken up by GC cells. In GC cells, the interaction between HGF and c-Met (mesenchymal-epithelial transition factor) activates downstream signaling pathways including Ras-Raf-MAPK and PI3K/AKT/mTOR. This activation leads to the phosphorylation and activation of MAPKs, allowing them to translocate into the cell nucleus and modulate the expression of c-Myc [67, 68]. In conclusion, MSCs facilitate the upregulation of c-Myc expression in GC cells by secreting IL-8 and HGF, thereby enhancing the expression of PD-L1 in GC cells.

**Fig. 4:**
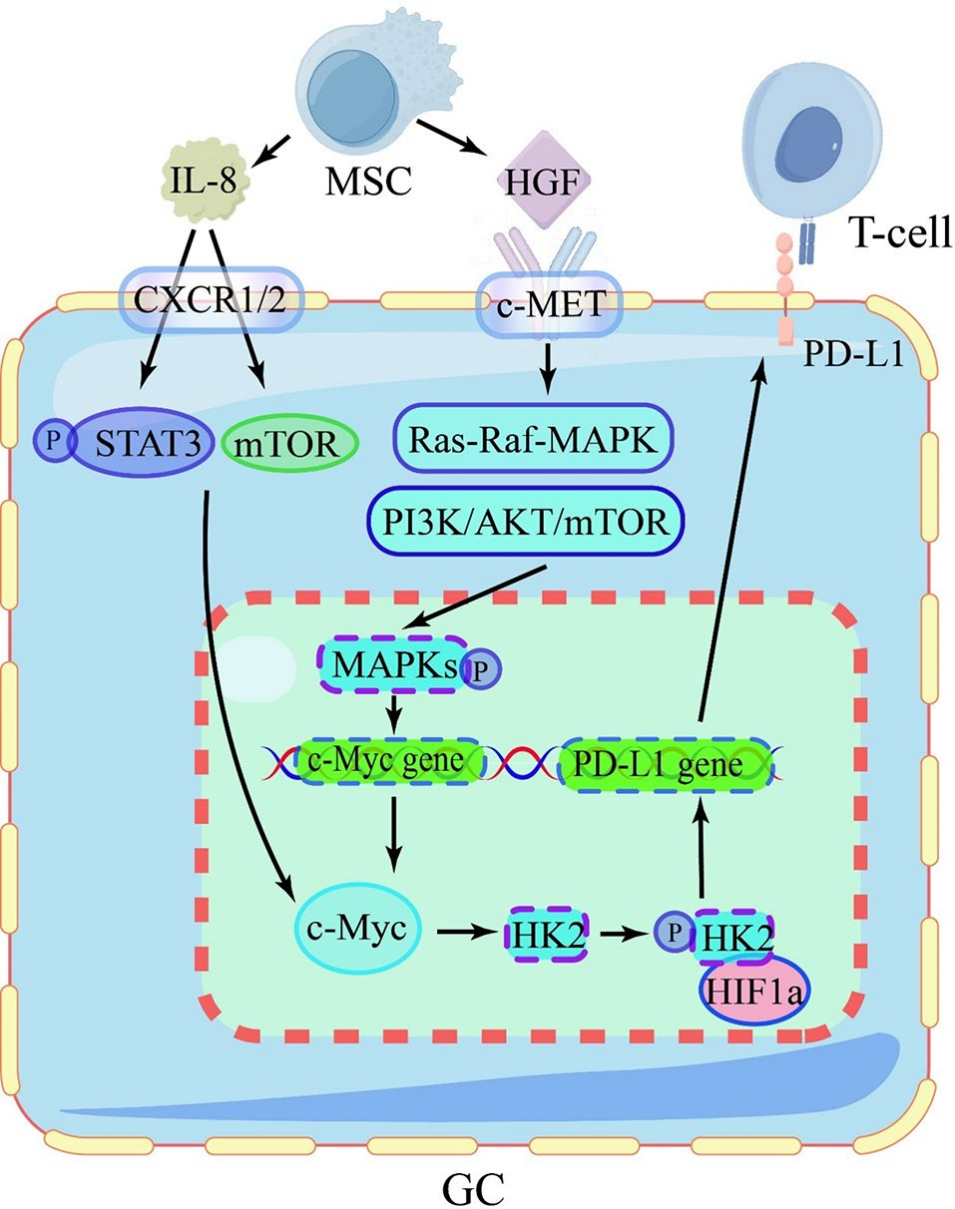
MSCs promotes immune escape of GC cell

In GC cells, c-Myc exerts a stimulatory effect on the upregulation of glucose transporter genes (glucose transporter Glut1, HK2, PKM2, LDHA, and PDK1), thereby facilitating a heightened metabolic breakdown of glucose into tricarboxylic acids and pyruvate, culminating in the ultimate conversion to lactate [69]. Previous studies have demonstrated that lactate can induce activation of the NF-κB signaling pathway in MSCs. Additionally, it has been observed that lactate-primed MSCs promote migration, proliferation, and PD-L1 expression in GC cells [70]. Building on these findings, it is postulated that lactate may stimulate HGF secretion by activating the NF-κB signaling pathway in MSCs, thereby inducing upregulation of c-Myc expression in GC cells(Fig. 4). However, concrete evidence supporting this hypothesis is currently lacking, highlighting a potential direction for future research.

Moreover, in vitro experiments and clinical sample tests have both shown that MSCs upregulating PD-L1 in GC cells can lead to increased Rad51 expression [71]. Rad51 is a key protein involved in homologous recombination, which is more active in cancer cells [72]. The enhancement of homologous recombination facilitates heightened recognition and response to double-strand breaks in GC cells, consequently diminishing the efficacy of cisplatin treatment [71].

In summary, MSCs can induce the upregulation of PD-L1 in GC cells via c-myc. Concurrently, as more GC cells evade the immune response, they generate higher levels of lactate, further stimulating MSC activity. This interaction highlights novel targets for immune checkpoint blockade in GC therapy. Moreover, the elevated PD-L1 expression induced by MSCs in GC cells exacerbates resistance to therapies like cisplatin, potentially guiding the development of more efficacious combination treatments.

The secreted IL-8 by MSCs binds to CXCR1/2 on GC cells, activating downstream STAT3 and mTOR pathways, thereby increasing C-myc levels; The secreted HGF by MSCs binds to c-MET on GC cells, activating the Ras-Raf-MAPK and PI3K/AKT/mTOR pathways, leading to the activation of MAPKs and upregulation of the C-myc gene; C-myc binds to HIFa to activate the PD-L1 gene, thereby increasing the expression of PD-L1.

### Tumor-associated neutrophils (TANs)

Neutrophils, integral components of the myeloid lineage, play pivotal roles in inflammation, tissue injury repair, and host defense against microbial invasion [73]. Recently, neutrophils have been implicated in the pathogenesis of various cancers, including GC, as they accumulate within the TME and foster an immunosuppressive and pro-tumor niche, thereby promoting cancer progression [74]. Neutrophils present in the TME, known as tumor-associated neutrophils (TANs), can exhibit either an anti-tumorigenic phenotype referred to as the “N1 phenotype” or a pro-tumorigenic phenotype known as the “N2 phenotype” [75, 76].

**Fig. 5:**
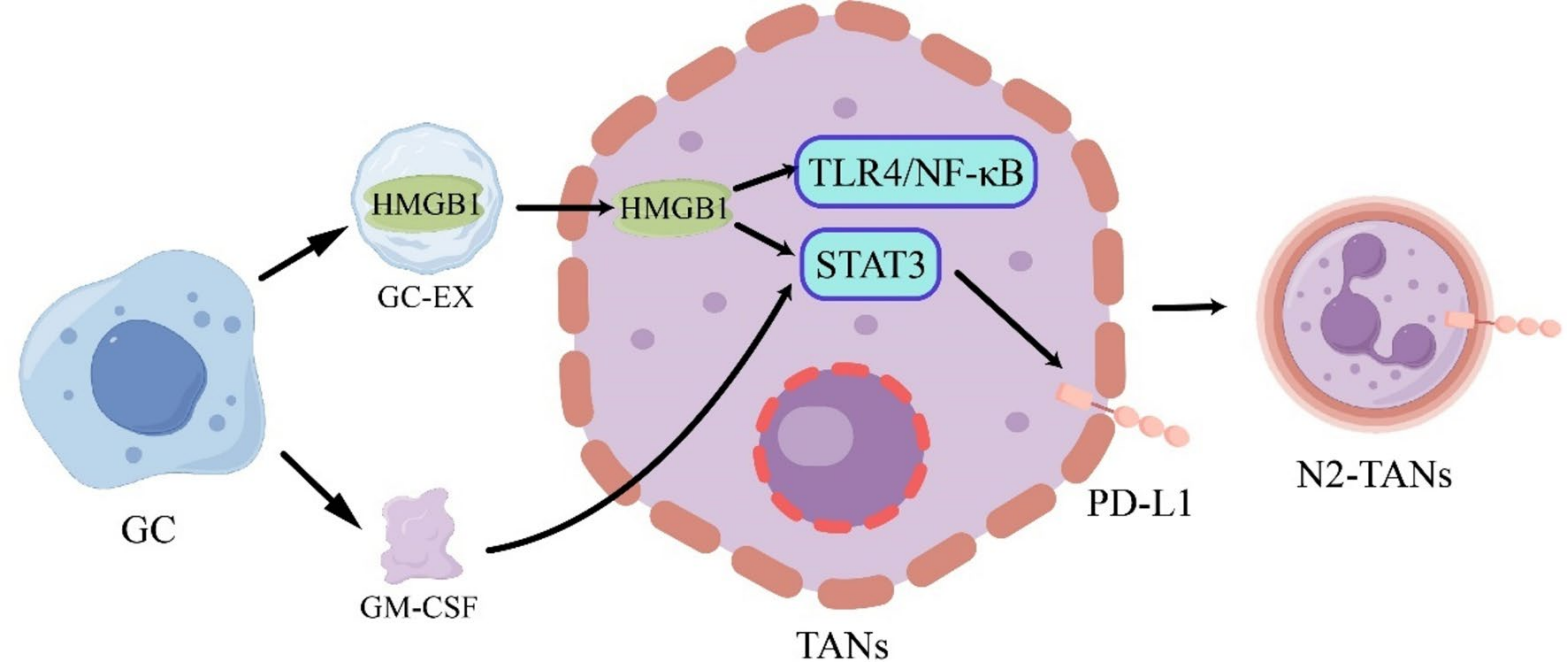
GC induces an increase in PD-L1 expression in TANs

N2-TANs secrete a diverse array of pro-angiogenic factors, facilitating tumor angiogenesis, while also exerting inhibitory effects on T cell and NK cell proliferation and function. Simultaneously, they attract regulatory T cells and macrophages, promoting tumor progression and metastasis [77–79]. In GC, various factors can influence neutrophil differentiation, with GC-Ex identified as promoting neutrophil polarization toward the N2 phenotype. In detail, GC-Ex can transport HMGB1 to neutrophils, thereby inducing N2 polarization of neutrophils through TLR4/NF-κB signaling transduction [80]. Shi et al. further elucidated that GC-Ex-mediated HMGB1 transport can lead to the upregulation of PD-L1 expression on neutrophils via the STAT3 signaling pathway [81]. Additionally, Wang et al. discovered that GC cells can release granulocyte-macrophage colony-stimulating factor (GM-CSF), which activates neutrophils to induce PD-L1 expression via the JAK/STAT3 signaling pathway (Fig. 5) [74]. This PD-L1/PD-1 interaction subsequently results in the generation of N2-TANs that suppress T cell immunity. Moreover, tumor-activated neutrophils also contribute to the modulation of the antitumor immune response of natural killer (NK) cells through the PD-L1-dependent cell-cell interaction mechanism [82].

In summary, under the influence of GC cells, there is an elevation in the expression of PD-L1 on TANs, consequently promoting the generation of a greater proportion of N2-type TANs. Treatment with PD-L1 targeting therapy may potentially halt or slow down this process, thereby regulating the ratio of N1 and N2 type cells, although further experimental validation is still required.

GC cells secrete GM-CSF and GC-EX containing HMGB1 to enter TANS cells, activating the STAT3 pathway to increase the expression level of PD-L1; HMGB1 can activate the TLR4/NF-κB signaling pathway, promoting the differentiation into N2-TANs.

### Tumor-associated macrophages (TAMs)

Macrophages are a versatile immune cell with diverse functions, including regulation of tissue homeostasis, defense against pathogens, and promotion of wound healing [83]. Macrophages residing within TME, also known as tumor-associated macrophages (TAMs), undergo differentiation into two distinct forms: M1 and M2.

Upon stimulation with inflammatory responses or microbial products such as lipopolysaccharides (LPS) and interferon-gamma (IFN-γ), TAMs can differentiate into the M1-like TAMs [84]. M1-like TAMs exhibit increased production of pro-inflammatory cytokines such as IL-12, IL-23, and TNF-α, thereby inhibiting tumorigenesis [85].

Additionally, TAMs can also differentiate into the M2 phenotype in response to anti-inflammatory cytokines like IL-4, IL-10, and transforming growth factor-beta (TGF-β). M2-like TAMs secret more anti-inflammatory cytokines, such as IL-10 and transforming growth factor-beta (TGF-β) [86]. They also express high levels of scavenger receptors, such as CD206 (mannose receptor), and secrete factors involved in tissue repair and remodeling. Importantly, M2-like TAMs play a pivotal role in immunosuppressive and tumorigenic phenotypes [83]. And the decrease of M1 macrophages and the increase of M2 macrophages in advanced tumors are markers of immunosuppression [87].

In GC, the expression of PD-L1 is associated with macrophages, and it shows a progressive increase with the density of macrophages [88]. Next, we will introduce the relationship between PD-L1 and macrophages in two parts.

#### M1-like TAMs

**Fig. 6:**
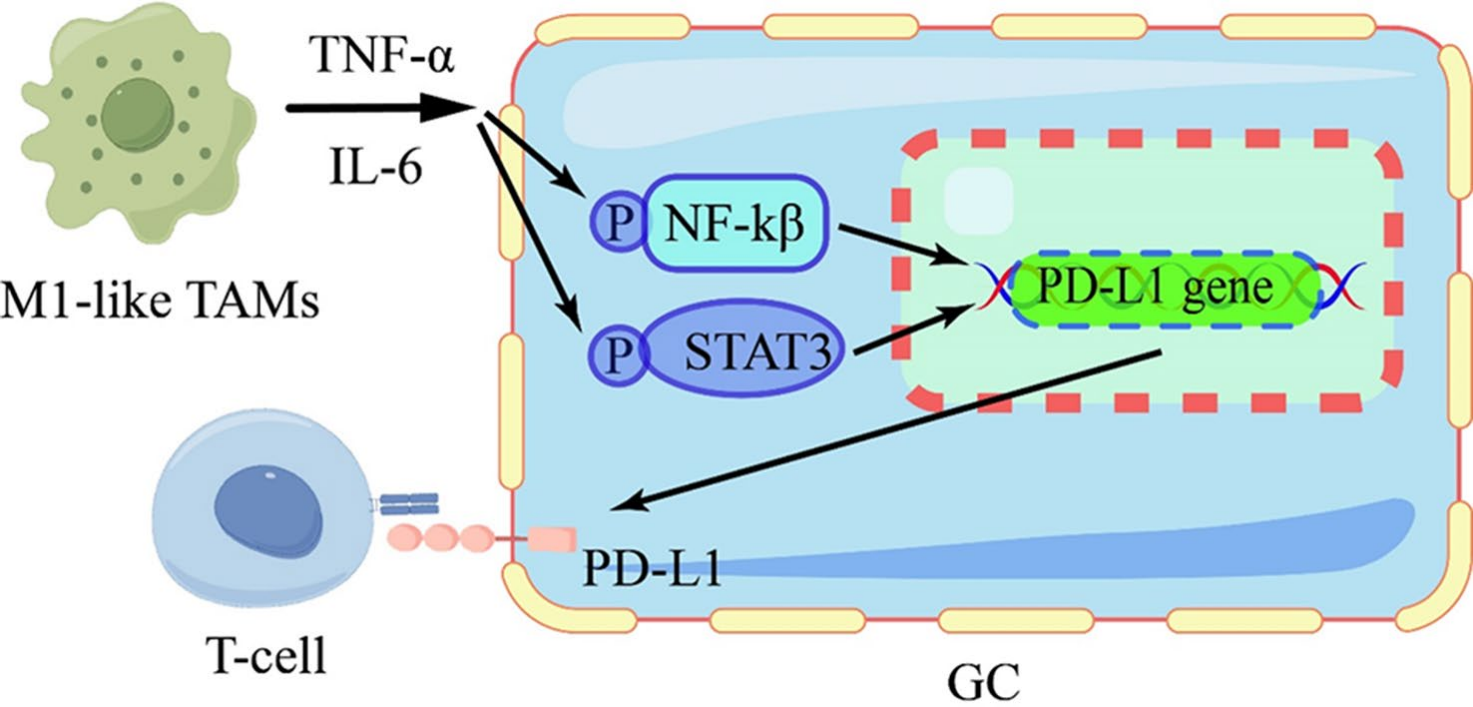
M1-like TAMs promotes immune escape of GC cell

The dynamic interplay between PD-L1 and M1-like TAMs significantly influences anti-tumor immunity. M1-like TAMs recruit CD8 + T cells to eliminate GC cells through the CXCL9, CXCL10, CXCL11/CXCR3 axis. Meanwhile, CXCL9, CXCL10, and CXCL11 can also upregulate the expression of PD-L1, enabling GC cells to evade CD8 + T cell-mediated cytotoxicity [89]. Additionally, M1-like TAMs have been shown to secrete IL-6 and TNF-α, leading to the induction of PD-L1 expression through activating the NF-kB and STAT3 signaling pathways in GC cells (Fig. 6). And the elevated levels of IL-6 and TNF-α are correlated with poor prognosis in GC [90]. Moreover, GC cell-derived CSF-2 promotes TAMs survival and polarization, ultimately leading to TAMs differentiation towards M1-like TAMs [91]. And CSF-2 can also induce increased secretion of CXCL8, which contributes to an immunosuppressive microenvironment via induction of PD-L1^+^ TAMs. Notably, higher levels of CXCL8^+^ macrophages and increased expression of PD-L1 are related to poor prognosis among patients with GC. However, it remains unclear about the mechanism CSF-2 promotes CXCL8 secretion and CXCL8 regulates PD-L1 expression [92]. Meanwhile, M1-like macrophage-derived exosomes carrying miR-16-5p have been demonstrated to inhibit PD-L1 expression and trigger T cell activation by promoting IL-2, TNF-α, and INF-γ expression, resulting to inhibition of GC [93]. The PD-L1/PD-1 blockades serve as highly effective therapy for GC subtypes characterized by abundant M1-type TAMs [89].

M1-like TAMs release IL-6 and TNF-α, activating the NF-kB and STAT3 signaling pathways in GC cells, resulting in increased PD-L1 expression.

#### M2-like TAMs

M2-like TAMs have been implicated in the TME due to their immunosuppressive and pro-tumorigenic properties. And GC patients exhibiting high infiltration of PD-L1 + and M2-like TAMs are associated with a poorer prognosis [94]. Meanwhile, high stromal PD-L1 expression levels have been linked to higher intertumoral densities of M2-like TAMs [95]. M2-like TAMs, when secreting IL-10, have the capacity to impair the function of CD8 + T cells and foster the creation of a tumor immune escape microenvironment. Furthermore, within an environment characterized by high infiltration of IL-10 + TAMs, there is a noticeable increase in the prevalence of PD-L1-positive cells. This implies a potential contribution of IL-10 to the upregulation of PD-L1 expression [96].

**Fig. 7:**
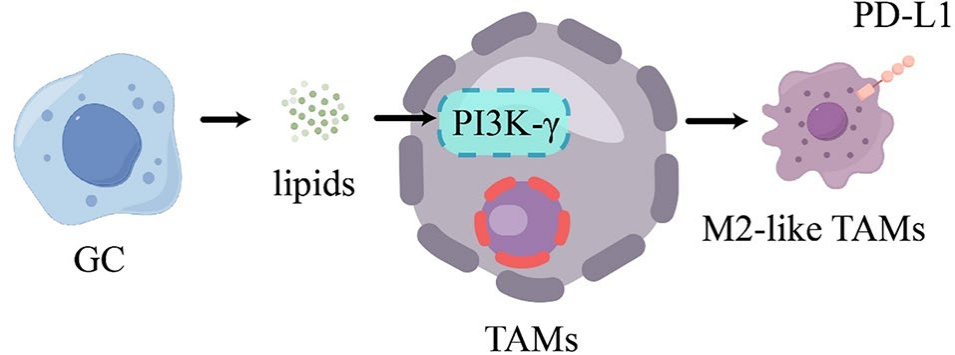
GC induces an increase in PD-L1 expression in M2-like TAMs

TAMs uptake lipids secreted by GC cells. This lipid accumulation prompts TAMs polarization towards the M2-like TAMs via activation of the PI3K-γ pathway. Meanwhile, M2-like TAMs with lipid accumulation elevate PD-L1 expression, leading to the suppression of anti-tumor T cell responses (Fig. 7) [97]. Selective inhibitors of PI3K-γ hold promise as a monotherapy or in combination with immune checkpoint blockade drugs for clinical trials in GC patients.

TAMs take up lipids released by GC, inducing their polarization towards M2-type TAMs through the PI3K-γ pathway, concomitant with upregulation of PD-L1 expression.

### Myeloid-derived suppressor cells (MDSCs)

Myeloid-derived suppressor cells (MDSCs) constitute a subset of myeloid cells that exhibit aberrant activation in pathological settings linked to diverse disease states, such as cancer, chronic inflammation, or stress. These cells play a critical role in promoting immune suppression, tumor angiogenesis, metastatic spread, and drug resistance, thereby driving the progression of cancer [98]. Clinically, the increased percentage of MDSCs is an independent risk factor for poor prognosis in GC [99]. Furthermore, the combination of PD-1 inhibitor and apatinib modulates the tumor microenvironment, enhancing anti-tumor effects in mice with GC. This therapeutic combination significantly elevates the ratio of CD4 + T cells and CD8 + T cells in the TME, concurrently reducing the proportion of MDSCs. This immunomodulatory effect contributes to enhanced treatment effectiveness and improved survival outcomes for GC patients [100].

**Fig. 8:**
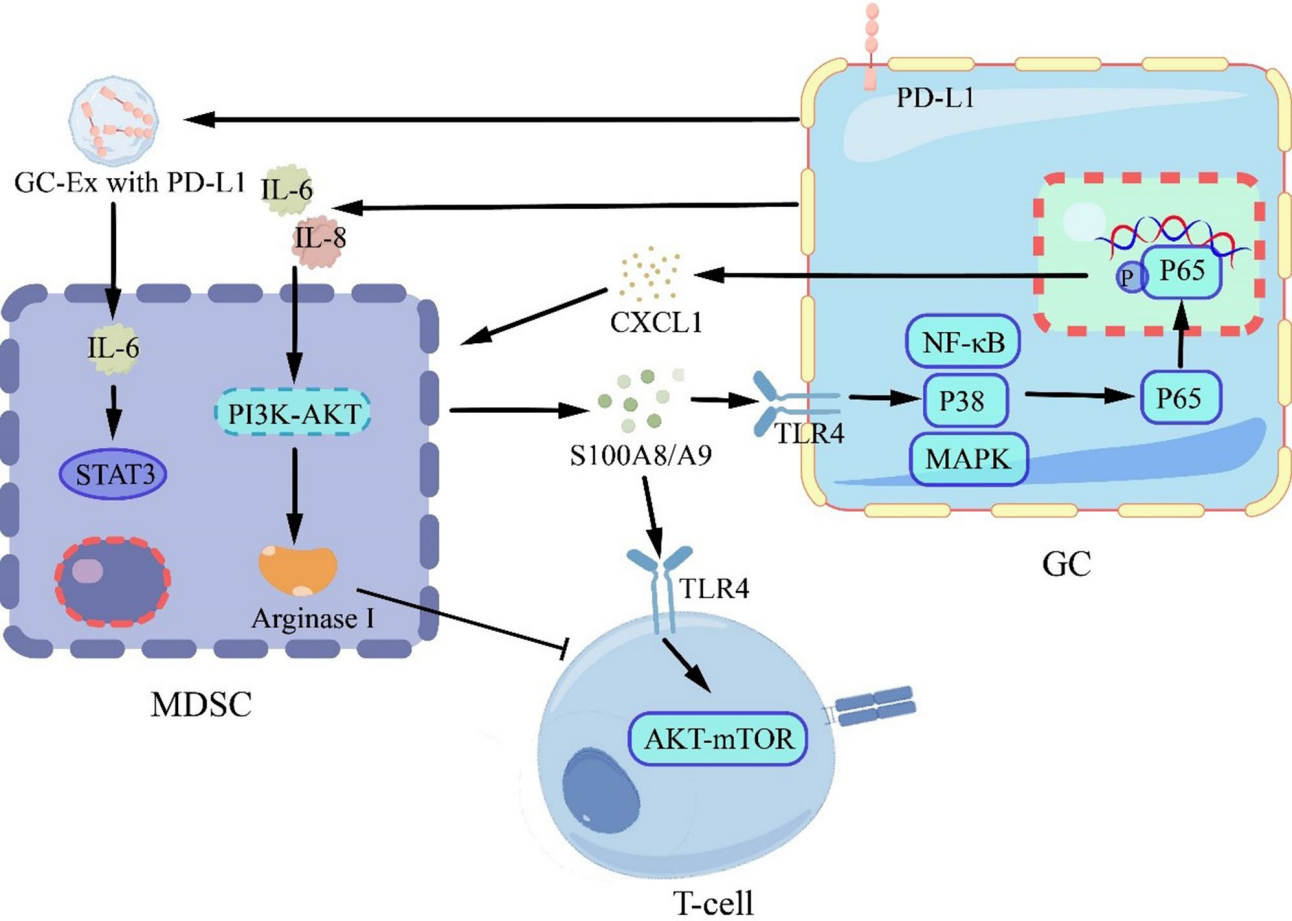
Interaction of MDSCs with GC Cells Leading to T Cell Suppression

Li, H’s team found that GC-Ex with PD-L1 promotes the proliferation of MDSCs via the activation of the IL-6/STAT3 pathway in a mice xenograft model [101]. And the overexpression of PD-L1 in GC also triggers the upregulation of chemokines like CXCL1 and osteopontin (Spp1), as demonstrated in mouse models [102]. CXCL1 promotes the recruitment of neutrophils and facilitates the migration of polymorphonuclear MDSCs, while Spp1 also contributes to MDSC recruitment and immunosuppressive activity [103]. In detail, there exists a positive feed-forward loop between CXCL1 and MDSCs. Polymorphonuclear MDSCs express S100A8 and S100A9, which are important members of the low molecular weight calcium-binding protein S100 family. S100A8/A9 induce the expression of CXCL1 in GC cells via the TLR4/p38 MAPK/NF-κB pathway. The expression of CXCL1 by GC cells recruits MDSCs through the CXCL1/CXCR2 axis. Additionally, MDSCs can contribute to CD8 + T cell exhaustion through the S100A8/A9-TLR4/AKT/mTOR signaling pathway [104]. Moreover, GC patient serum-derived IL-6 and IL-8 activate and induce MDSCs to express arginase I via the PI3K-AKT signaling pathway, which in turn inhibits the activity of CD8 + T cells(Fig. 8) [105]. Recently, a PD- L1 targeting high- affinity natural killer (t- haNK) cell line was designed, which are specialized haNK cells engineered to target PD- L1 expressing tumor cells via CAR. Moreever, PD-L1-t-hank cells also show high activity for lysing human MDSCs with high PD-L1 expression in vitro. However, there is no research on GC cells [106].

In summary, MDSCs not only contribute to GC progression by facilitating immune suppression and tumor angiogenesis but also intensify the immunosuppressive through positive feedback loops mediated by chemokines within GC cells highly expressing PD-L1.

GC-Ex with PD-L1 promoted MDSC expansion via activation of IL-6/STAT3 pathway. MDSCs produce S100A8/A9, which drives CXCL1 expression in GC cells through the TLR4/p38 MAPK/NF-κB pathway and contributes to CD8 + T cell exhaustion via the TLR4/AKT/mTOR cascade. These PD-L1-expressing GC cells secrete CXCL1 to promote MDSC migration. Upon receiving IL-6 and IL-8 from GC cells, MDSCs activate PI3K-AKT signaling to express arginase I, thereby suppressing T cell function.

### Mast cells

**Fig. 9:**
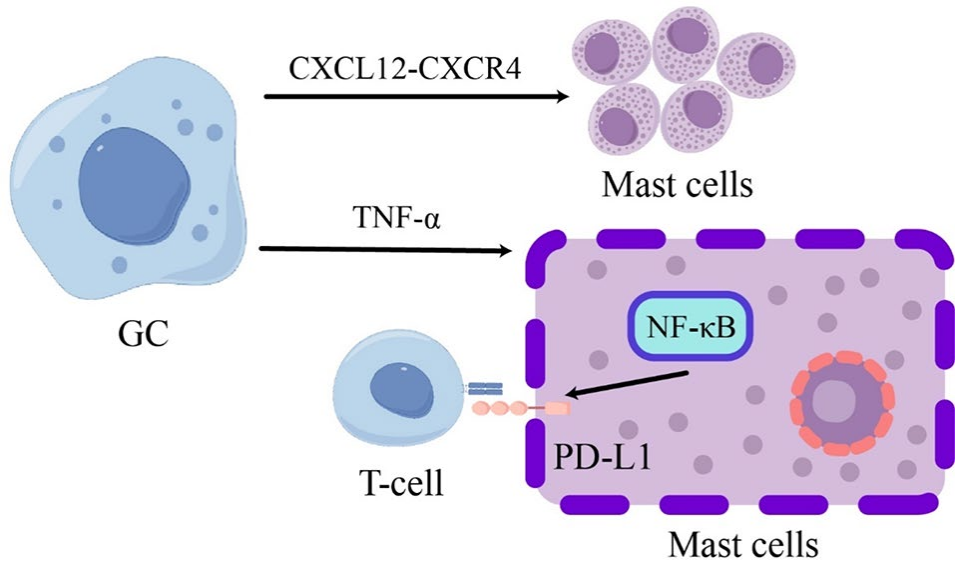
Interaction of mast cells with GC Cells Leading to T Cell Suppression

Mast cells, a type of immune cell derived from CD34 + and CD117 + pluripotent stem cells in bone marrow, are widely distributed around various tissues including epithelial cells, fibroblasts, blood and lymphatic vessels, and nerves. These cells are involved in diverse physiological and inflammatory processes including tumorigenesis [107]. The underlying mechanism could be attributed to a tumor-promoting microenvironment created by GC tumors that leads to the recruitment of mast cells into the tumor bed via CXCL12-CXCR4 interaction. Tumor-derived TNF-α activates the NF-κB pathway to induce PD-L1 expression on mast cells(Fig. 9). It also has been demonstrated that the accumulation of intratumoral mast cells is positively correlated with GC progression and poor clinical outcomes among patients [108].

The chemotaxis of mast cells into the GC microenvironment is facilitated by CXCL12-CXCR4 signaling, while tumor-derived TNF-α activates the NF-κB pathway to up-regulate PD-L1 expression. In the GC setting, mast cells suppress T-cell proliferation and function in a PD-L1-dependent manner, thereby promoting GC progression.

### Tumor-associated fibroblasts (TAFs)

Tumor-associated fibroblasts (TAFs), found near primary and metastatic tumors, produce extracellular matrix components and remodeling enzymes, promoting structural changes in the surrounding tissue that enhance tumor growth and metastasis [109]. Elevated expression of Lipoma preferred partner (LPP) protein in TAFs has been correlated with adverse prognosis in GC patients, while higher levels of PD-L1 expression were also observed in patients with increased LPP protein levels [110]. Moreover, research has also revealed that IL-8 originating from cancer-associated fibroblasts (CAFs) enhances the expression of PD-L1 in GC cells through the activation of the P38, JNK, and NF-κB pathways [111]. TAFs in the deep stroma exhibit high expression of Glypican-3, which are associated with poor prognosis and diminish the efficacy of PD-1/PD-L1 immune therapy [112].

## Other factors influencing PD-L1 expression

### Autophagy

Autophagy is an evolutionarily conserved, complex catabolic process that contributes to maintaining cellular homeostasis by degrading cytoplasmic constituents. During autophagy, macromolecules and damaged or unnecessary organelles in the cytoplasm are delivered to lysosomes for breakdown by hydrolytic enzymes into amino acids, nucleotides, sugars, fatty acids, and ATP [113]. Studies have revealed that blockade of this crucial process results in p62/SQSTM1 accumulation and subsequent activation of NF-κB pathway, leading to prominent upregulation of programmed death-ligand 1 (PD-L1) in GC [114].

### Lysine-specific histone demethylase 1 (LSD1)

LSD1, an evolutionarily conserved transcriptional corepressor, has been demonstrated to enhance the proliferation and metastasis of GC cells [115, 116]. Its expression negatively correlates with CD8 + T-cell recruitment and activation, leading to impaired anti-tumor immunity. In GC, LSD1 deletion reduces total cellular PD-L1 expression, decreasing exosome secretion containing PD-L1. These PD-L1-carrying exosomes directly inhibit T-cell activation and can be transferred to other cells, promoting immune evasion [117]. Various LSD1 inhibitors have been developed in the laboratory, showing promise in enhancing T-cell-mediated tumor killing and suppressing tumor growth by inhibiting PD-L1 expression [118, 119]. The relationship between LSD1 and PD-L1 in shaping the immune microenvironment of GC underscores the potential for targeted interventions to mitigate tumor-associated immunosuppression.

### Epithelial-mesenchymal transition (EMT)

EMT refers to the loss of polarity, tight junctions, and adhesion of epithelial cells induced by various factors, leading to the transformation of epithelial cells into interstitial-like cells. EMT can enhance cancer cell metastatic properties by increasing mobility, invasion, and resistance to apoptotic stimuli [120]. In TGFβ1-induced EMT tissues, TGFβ1 upregulated PD-L1 expression by activating NF-kB transcription. The expression of PD-L1 was significantly elevated in EMT-high cell lines compared to EMT-low cell lines [121]. It is worthy of exploration to utilize the interaction mechanisms between EMT and PD-L1 to enhance the effectiveness of immunotherapy and improve the treatment outcomes of GC patients.

### Melatonin

Melatonin, as an important indoleamine hormone, plays a crucial role in regulating circadian rhythms, anti-tumor activity, and immune function [122]. Melatonin has been demonstrated to inhibit the proliferation of GC cells through the modulation of the miR-16-5p against decapentaplegic homolog 3 pathway [123]. Additionally, recent studies have shown that melatonin suppresses the expression of PD-L1 in GC cells and TAMs. In GC, melatonin increases the levels of miR-20b-5p、miR-17-5p and miR-93-5p in extracellular vesicles derived from GC cells, resulting in a downregulation of PD-L1 expression [124]. The PD-L1 in this process is conjectured, yet empirical validation is lacking at present. In an experiment involving neck squamous cell carcinoma (HNSCC), the pretreatment of HNSCC cells with melatonin did not further enhance T cell activity when the PD-1/PD-L1 signaling was blocked by anti-PD-L1 antibodies. This suggests that the anti-tumor effects of melatonin in this context are mainly mediated by reducing the expression levels of PD-L1 [121]. However, similar experimental evidence has not been demonstrated in GC.

## Conclusion and prospects

The TME is a complex ecosystem involving various components such as surrounding cells, blood vessels, lymphatic vessels, and the extracellular matrix. In GC, a significant feature of the TME is the augmented expression of PD-L1 in multiple cell types, which impairs T cell function and promotes immune evasion. The initiating factors that lead to increased PD-L1 expression are diverse, primarily categorized as exogenous influences from pathogens and interactions among microenvironmental components during tumor progression.

In terms of pathogens, EBV and *H. pylori* have the capacity to induce GC. Even in the absence of tumor formation, long-term infection can create an inflammatory environment that results in increased PD-L1 expression, fostering an immune-suppressive microenvironment. During the onset and development of tumors, both EBV and *H. pylori* exhibit unique mechanisms that elevate PD-L1 expression in GC cells and other cells within the microenvironment. These mechanisms involve multiple signaling pathways and interactions between various cell types. Blocking a single pathway may not suffice, as other pathways may still lead to increased PD-L1 expression in the TME. Thus, the best approach for addressing PD-L1 expression induced by these pathogens is the eradication of the infectious agents.

Apart from exogenous factors like pathogens, interactions between cells within the GC-TME also contribute to increased PD-L1 expression. For instance, MSCs and M1-like TAMs can induce the upregulation of PD-L1 in GC cells. Mast cells and TANs, influenced by GC cells, also exhibit increased PD-L1 expression. Additionally, TANs and TAMs, possessing both pro-tumorigenic and anti-tumorigenic phenotypes, tend to differentiate more into pro-tumorigenic phenotypes and express PD-L1 within the TME. Interestingly, despite their anti-tumorigenic phenotype, M1-like TAMs can also elevate PD-L1 expression in GC cells, potentially indicating a mechanism by which the TME counteracts their tumor-suppressive functions.

The elevation of PD-L1 expression is a progressive phenomenon within the TME over time. The body employs immune mechanisms to combat tumor growth, while TME generate PD-L1 to counteract this immune response. Clarifying the mechanisms that lead to elevated PD-L1 expression in various cells presents an opportunity to identify effective targets for inhibiting or reducing PD-L1 levels in the TME.

## Data Availability

Not applicable.
